# Continuities in Emotion Lateralization in Human and Non-Human Primates

**DOI:** 10.3389/fnhum.2013.00464

**Published:** 2013-08-08

**Authors:** Annukka K. Lindell

**Affiliations:** ^1^School of Psychological Science, La Trobe University, Melbourne, VIC, Australia

**Keywords:** perception, expression, asymmetry, hemisphere, face, chimpanzee, macaque, baboon

## Abstract

Where hemispheric lateralization was once considered an exclusively human trait, it is increasingly recognized that hemispheric asymmetries are evident throughout the animal kingdom. Emotion is a prime example of a lateralized function: given its vital role in promoting adaptive behavior and hence survival, a growing body of research in affective neuroscience is working to illuminate the cortical bases of emotion processing. Presuming that human and non-human primates evolved from a shared ancestor, one would anticipate evidence of organizational continuity in the neural substrate supporting emotion processing. This paper thus reviews research examining the patterns of lateralization for the expression and perception of facial emotion in non-human primates, aiming to determine whether the patterns of hemispheric asymmetry that characterize the human brain are similarly evident in other primate species. As such, this review seeks to enhance understanding of the evolution of hemispheric specialization for emotion, using emotion lateralization in non-human primates as a window through which to view emotion lateralization in humans.

For much of the past 150 years, hemispheric lateralization has been considered an exclusive characteristic of the human brain. However, where once cerebral asymmetry was thought “the most imposing difference between man and animal,” (Pruner-Bey, 1865, p. 558), it is increasingly recognized that cerebral asymmetries present throughout the animal kingdom (e.g., Rogers and Andrew, 2002; Ocklenburg and Güntürkün, 2012), across both vertebrates (e.g., Corballis, 2009) and invertebrates (e.g., Taylor et al., 2010). Far from being exclusive to humans, species from amphibians (e.g., Vallortigara, 2006) and fish (e.g., Lippolis et al., 2009), to reptiles (e.g., Csermely et al., 2010), birds (e.g., Rogers, 2008), and mammals (e.g., Levy, 1977), evidence asymmetries in brain and behavior, suggesting that lateralization is a fundamental principle of nervous system organization. Despite this, the popularity of the presumption that lateralization was restricted to humans has limited the integration of research across human and non-human species. Such integration is needed to help shed light on the phylogeny of hemispheric asymmetry.

The notion that the human brain is functionally lateralized was first mooted by Broca (1861) following his observation that left hemisphere insult was intimately linked with language impairment. Whilst language is undoubtedly the paradigmatic lateralized function, it is not alone: emotion processing also exhibits clear evidence of functional lateralization in humans. Although the precise nature of the lateral division of emotion remains somewhat contentious (for review please see Demaree et al., 2005; Harmon-Jones et al., 2010; Rutherford and Lindell, 2011), the right hemisphere is widely regarded to play the dominant role in emotion processing. This lateralization of function confers efficiency benefits, removing redundancy associated with the reduplication of function, preventing conflict between the hemispheres, and facilitating performance of multiple simultaneous tasks (e.g., Rogers et al., 2004; Reddon and Hurd, 2009; Salva et al., 2012).

The purpose of emotion is to facilitate adaptive behavior and decision making in response to salient events (Davidson et al., 2007). As such, emotion is vital to survival. Emotional expressions play a powerful communicative role for we convey emotional states to others via the stereotypic posturing of facial features (Leopold and Rhodes, 2010). Emotional expressions are thus a key component of social interactions, indicating the likely future behavior of the displaying animal (Andrew, 1963), communicating intentions and desires, and influencing others’ emotional states. In highly social species like primates, the ability to decode emotional facial expressions efficiently and effectively confers significant evolutionary advantage (e.g., efficient threat detection aids self-preservation by prompting a fight/flight response). Given the importance of emotion to primate survival, a growing body of research in affective neuroscience is dedicated to shedding light on the neural substrates supporting emotion processing, and providing clues concerning phylogenies in human and non-human primates.

Presuming that human and non-human primates evolved from a shared ancestor (Stewart and Disotell, 1998), one would expect evidence of organizational continuity in the neural substrates supporting emotion processing. This paper thus reviews research examining patterns of lateralization for the expression and perception of facial emotion in non-human primates, assessing whether the characteristic right hemisphere dominance for emotion processing seen in humans is similarly evident in other primate species. As such, this review seeks to enhance understanding of the evolution of hemispheric specialization for emotion by using emotion lateralization in non-human primates as a powerful window through which to view emotion lateralization in humans.

## Emotion Lateralization in Humans

Following Broca’s (1861) discovery that language was functionally lateralized in the human brain, Hughlings-Jackson (1874/1915) reported that emotion was also lateralized. Based on his repeated clinical observation that right hemisphere damage led to deficits in producing and perceiving emotion, Hughlings-Jackson proposed that emotion was lateralized to the right hemisphere (*the right hemisphere hypothesis*; see also Demaree et al., 2005, for review of an alternate model: *the valence hypothesis*). Subsequent observations of clinical patients offer further support for the right hemisphere hypothesis, demonstrating that damage to the right hemisphere compromises both the perception (e.g., Bowers et al., 1985; Borod et al., 1992) and the production of emotion (e.g., Borod et al., 1986; Blonder et al., 1993). These clinical findings converge with the results of behavioral investigations in neuro-typical populations (e.g., Wittling and Roschmann, 1993; Calvo and Avero, 2008), confirming right hemisphere dominance for emotion processing.

**Figure 1:**
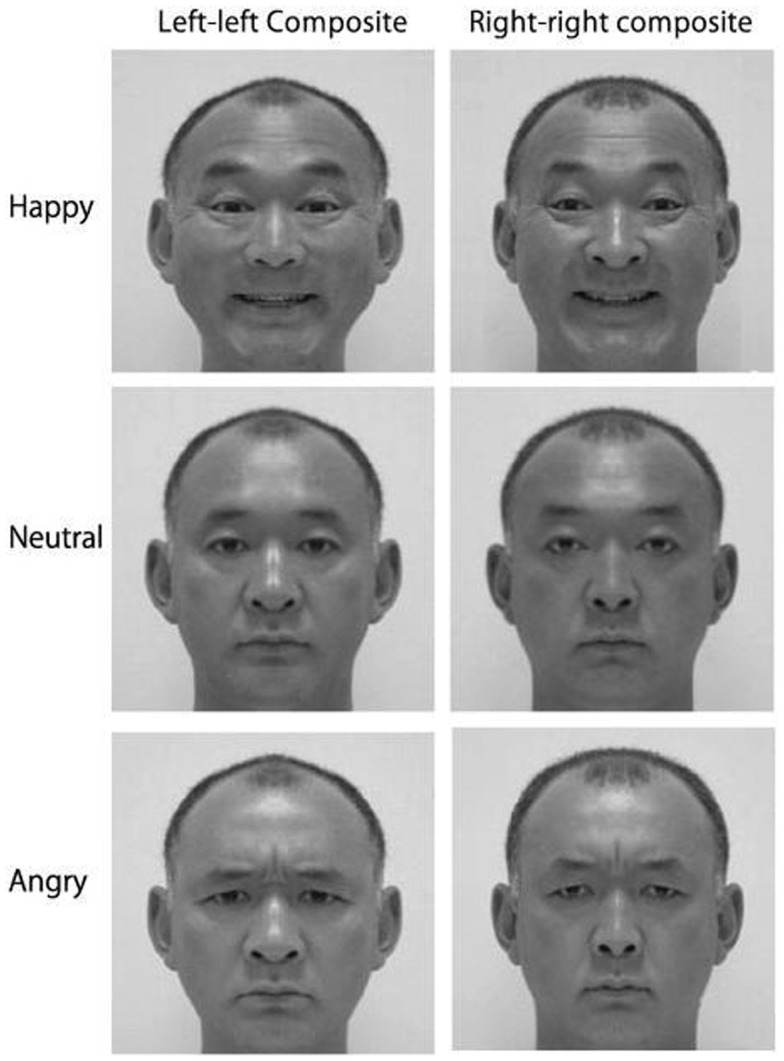
**Examples of mirrored chimeric face stimuli; people tend to select the left–left chimeras as showing stronger emotion than the right–right chimeras (Reprinted from Okubo et al., 2013, Copyright with permission from Elsevier)**.

The cortical asymmetry evident for emotion processing leads to an expressional asymmetry: though we are rarely conscious of it, human emotional expressions are asymmetric. Thus, whether we are grinning or grimacing, we show stronger emotion on the left side of the face (e.g., Indersmitten and Gur, 2003). As the lower two-thirds of the face is innervated contralaterally (Rinn, 1984; Patten, 1996), with bilateral projections increasing in the upper face (Matsumoto and Lee, 1993), the left side of the face is predominantly controlled by the right hemisphere. Given the right hemisphere’s dominance for emotion control (e.g., Demaree et al., 2005), the muscles on the left side of the face move more than those on the right side of the face during emotional expression (e.g., Dimberg and Petterson, 2000), consequently producing a more intense expression. Not surprisingly then, Borod’s (1993) review of 47 studies examining facial expression asymmetries in the normal population concluded that the left hemiface produces more intense emotional expressions than the right hemiface. As the left side of the face is more emotionally expressive, chimeric faces composed of mirrored left-cheeks are perceived as showing stronger emotion than mirrored right-cheek composites (Sackeim et al., 1978; please refer to Figure 1), and left cheek portraits appear more emotionally expressive than those showing the right cheek (e.g., Nicholls et al., 2002; see Lindell, in press, for review).

The observation that human facial expressions are asymmetric is not new. Darwin (1872) first reported that for expressions such as “sneering defiance,” the upper lip is “raised on one side alone in sneering at or defying any one… (although) movement being confined to one side may not be an essential part of the expression, but may depend on the proper muscles being incapable of movement excepting on one side,” (p. 253). In Darwin’s view, human expressions shared much with those of other animals, contrary to facial anatomist Sir Charles Bell’s claims that God designed humans with unique facial muscles to express uniquely human emotions (Matsumoto and Ekman, 2008); by adopting a comparative, evolutionary approach, Darwin noted commonalities between human expressions and those of our “semi-human progenitors,” (p. 254). Unfortunately however, this comparative, evolutionary study of facial expressions has attracted little interest, as researchers from Andrew (1963) to Leopold and Rhodes (2010) lament.

Beyond asymmetries in the expression of emotion, the right hemisphere’s superiority for emotion processing also manifests in asymmetries when perceiving emotion. Clinical research confirms that damage to the right hemisphere impairs the ability to identify and discriminate facial emotions, whereas damage restricted to the left hemisphere does not affect emotion recognition (e.g., Adolphs et al., 1996). The emotion perception impairment resulting from right hemisphere damage is not restricted to emotion conveyed via the facial channel: right hemisphere damage also compromises the perception of emotional words (Borod et al., 1998), and impairs the ability to interpret emotional prosody (Tucker et al., 1977). Consistently, anesthetizing the right hemisphere causes patients to judge facial emotional expressions as less intense than when the left hemisphere is anesthetized (Ahern et al., 1991). As such, the clinical data clearly implicate a dominant right hemisphere role in emotion perception.

**Figure 2:**
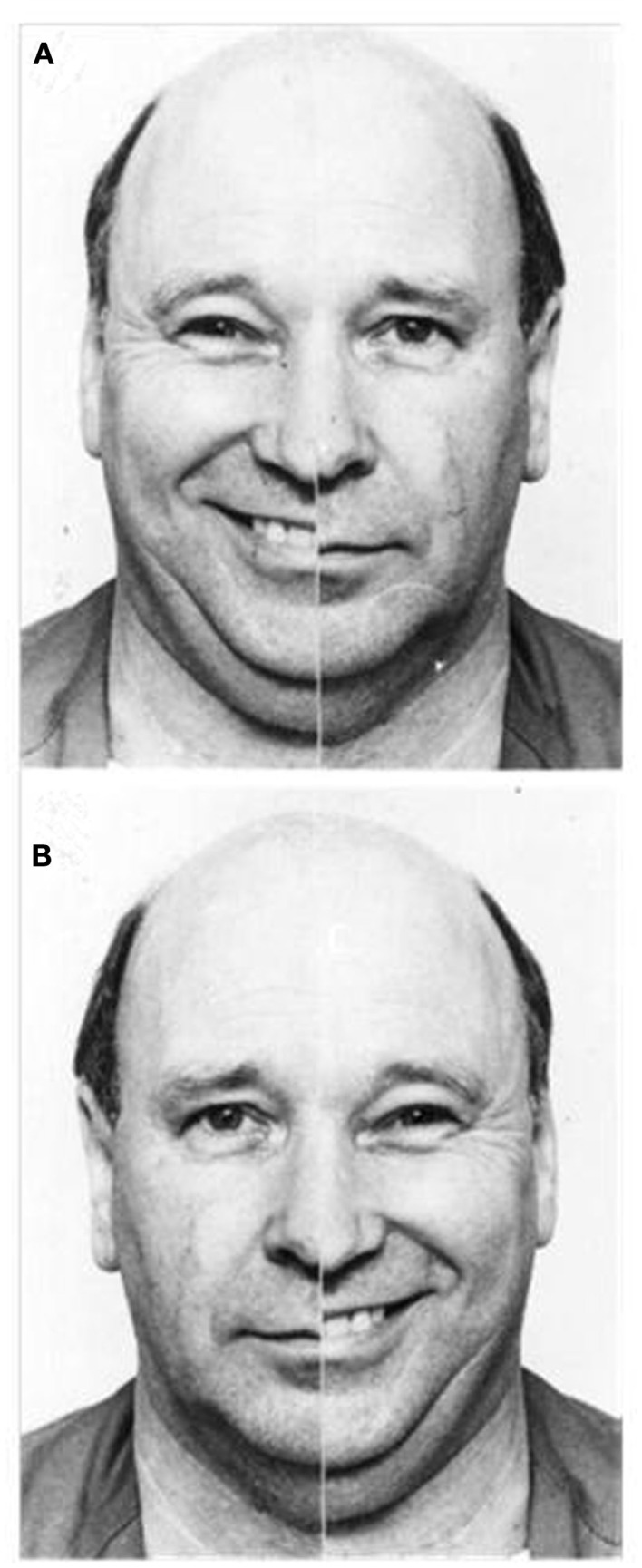
**Examples of emotional/neutral chimeric face stimuli; people tend to select the image in which emotion is presented in the left visual field (A) as more expressive than the identical image reversed to show emotion in the right visual field (B) (Reprinted from Failla et al., 2003, Copyright with permission from Elsevier)**.

Data from both imaging and behavioral studies similarly highlight strong right hemisphere involvement when we perceive emotion. Across a range of paradigms and communicative channels, functional imaging research indicates that perceiving emotion expressed via faces (Gorno-Tempini et al., 2001), prosody (Wildgruber et al., 2005), and even music (Blood et al., 1999), prompts more pronounced activation in the right than left hemisphere. Results of behavioral investigations appear congruent, with participants exhibiting a left visual field (right hemisphere) advantage for recognizing emotional expressions in faces (Ley and Bryden, 1979), leading to a perceptual bias when viewing emotional/neutral chimeric faces: chimeras showing emotion in the viewer’s left visual appear more emotionally expressive than chimeras showing emotion in the viewer’s right visual field (Failla et al., 2003; please refer to Figure 2). This emotional asymmetry is similarly evident for audition, with participants showing a left ear (right hemisphere) advantage for recognizing emotional words (Sim and Martinez, 2005), and emotional tones (Bryden et al., 1982).

In sum, the evidence indicates that the right hemisphere plays a crucial role in emotion processing in humans. Across a broad range of research paradigms, including clinical, functional imaging, and behavioral investigations, the data highlight the importance of the right hemisphere in both the expression and perception of facial emotion. The question to which we now turn is whether the hemispheric asymmetry for emotion processing present in humans is similarly evident in non-human primates, thus suggesting conservation across phylogeny.

## Emotional Expression in Primates

**Figure 3:**
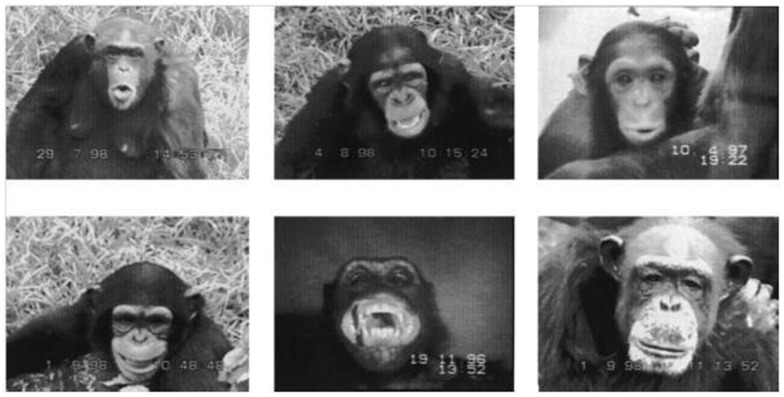
**Examples of chimpanzee facial expressions**. Top row from left to right: pant-hooting, play face, silent pout. Bottom row from left to right: silent bared-teeth display, staring bared-teeth scream face, neutral face (Reprinted from Fernández-Carriba et al., 2002a, Copyright with permission from Elsevier).

Across the animal kingdom, species convey information regarding emotional state via different communicative channels (e.g., vision, olfaction, audition). A visual means of emotional expression is widely used by diurnal social mammals, including primates (Tate et al., 2006); in non-human primates, facial expressions of emotion are typically accompanied by vocalization (e.g., Hauser, 1993). The facial expression of emotion necessitates exquisite facial mobility: to communicate effectively, faces must be configurable into a variety of postures (please refer to Figure 3). Not surprisingly then, facial mobility has increased over the course of primate evolution (Andrew, 1963), facilitating a greater variety and more precise expressional displays that serve to reduce uncertainty about behavioral intent and thus promote social cohesion (Parr et al., 2007a). Such displays offer more specific information about the probable future behavior of the displaying animal, conferring an evolutionary advantage for highly social animals, hence the trend toward increased facial mobility across primates’ evolutionary history (Andrew, 1963).

Of all the mammals, primates are argued to possess the most complex facial musculature, thus enabling the most intricate facial displays (Burrows, 2008). Though it was once thought that the complexity of primates’ facial musculature increased as you traveled up the phylogenetic tree (i.e., low complexity for galagos, lorises, and lemurs; high complexity for chimpanzees and humans; Huber, 1931), more recent research indicates that the number of facial muscles in the lower primates (17 muscles in galagos and lemurs; Burrows, 2008) is much closer to that of the higher primates (23 muscles in humans and chimpanzees; Burrows et al., 2006) than previously estimated. Looking particularly at humans and our closest relative, the chimpanzee, Burrows et al.’s anatomical work confirms that the 23 facial muscles present in humans are *all* present in chimpanzees. Indeed, comparative research confirms that intramuscular electrical stimulation of the individual facial muscles prompts functionally similar changes in appearance in both chimpanzees and humans (Waller et al., 2006). Not surprisingly then, this similarity in musculature means that the basic repertoire of facial expressions available to humans is similar to that observed in chimpanzees (Parr et al., 2007a,b).

Studies of reflexive facial expressions in response to different tastes offer evidence of expressional congruity across primate species, with salty and bitter tastes prompting aversive emotional expressions whereas sweet tastes elicit positive emotional expressions (Erickson and Schulkin, 2003). These emotional responses to sweet and bitter tastes are homologously observed across primate species, including lemurs, Old and New World monkeys, great apes, and humans (Steiner et al., 2001). Critically, the degree of similarity in pattern of expression closely reflects phylogenetic proximity: human expressions are more similar to those of the great apes than either species’ expressions are to Old or New World monkeys. Given that humans and great apes are thought to have shared a common ancestor within the last 10–20 million years (Stewart and Disotell, 1998), and their hominoid ancestors both diverged from Old World monkeys 20–40 million years ago (Arnason et al., 1996), the congruity in expression for more closely related species appears apposite and is consistent with cytoarchitectural differences in oro-facial motor cortex between the hominoid species (humans and great apes) and Old World monkeys (Sherwood et al., 2004).

## Emotion Lateralization in Non-Human Primates: Expression

Analysis of facial expression asymmetries in non-human primates suggests that the right hemisphere/left hemiface emotion bias evident in human expression has its precursors in non-human primate evolution. Across a variety of non-human primate species, including rhesus macaques (e.g., Hauser, 1993), baboons (e.g., Wallez and Vauclair, 2011), and chimpanzees (e.g., Fernández-Carriba et al., 2002a,b), the left side of the face produces more pronounced emotional expressions, with the left side of the mouth opening wider and mobilizing earlier during emotional calls. Given that the left side of the face/mouth is contralaterally controlled by the right hemisphere in both human and non-human primates (e.g., Patten, 1996; Morecraft et al., 2001), more pronounced expressivity in the left side of the face is taken to index right hemisphere dominance for emotional expression.

When Hauser (1993) assessed asymmetries in adult rhesus macaques’ facial expressions (fear grimace, copulation grimace, open mouth threat, ear flap), results indicated that the left side of the face was both more expressive and more mobile. For example, when producing a fear grimace, there were more expression folds on the left side of the face, and the left corner of the mouth reached a higher position, than the right. As the left side of the face is predominantly controlled by the right hemisphere in macaques (e.g., Morecraft et al., 2001), Hauser’s findings suggest right hemisphere dominance for emotion control in macaques. Moreover, analysis of the timings of expression emergence indicated that the left side of the face commenced movement first (fear grimace, open mouth threat) and maintained the expression for longer (copulation grimace) than the right side. This finding has subsequently been replicated by Hauser and Akre (2001), with both infant and adult macaques showing earlier initiation of emotional expressions on the left side of the face, again implicating a greater right hemisphere role in emotional control.

**Figure 4:**
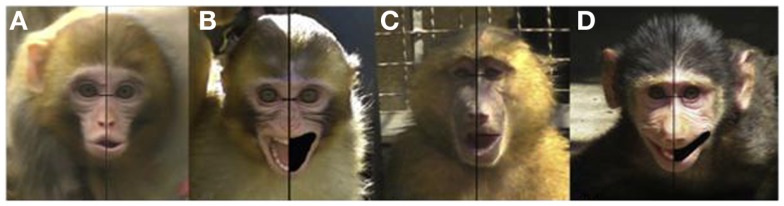
**Examples of asymmetries in expressions in infant macaques [(A) cooing; (B) screaming] and infant baboons [(C) moaning; (D) gecking] (Reprinted from Wallez and Vauclair, 2012, Copyright with permission from Elsevier)**.

This left side bias has also been reported for screeching in adult baboons (Wallez and Vauclair, 2011, in press), with recent research confirming that it is evident early in development, being present in both infant macaques (cooing) and infant baboons (gecking; Wallez and Vauclair, 2012; please refer to Figure 4). Given that both baboons and macaques are Old World monkey species, these data suggest that the right hemisphere’s specialization for the control of emotional expression must have emerged early in primate evolution (at least 30–40 million years ago, Boyed and Silk, 2000) and was conserved in later-evolving primate species, including both chimpanzees and humans.

Fernández-Carriba et al.’s (2002a,b) research on chimpanzee facial expressions again implicates a strong right hemisphere role in emotional expression. They made natural observations of chimpanzees interacting over two 9-month periods, and recorded/coded five categories of facial expressions (pant-hooting, play face, silent pout, silent bare-teeth display, staring bared-teeth scream face). Quantitative analysis of the resulting 183 facial images indicated that the left hemi-mouth subtended a larger area than the right (hooting, play, silent bared-teeth), and extended laterally more than the right (hooting, silent bared-teeth, scream face). As such, these findings appear consistent with a right hemisphere dominant functional asymmetry for emotion control.

Importantly, research comparing oro-facial asymmetries in non-human primates’ emotional and intentional communicative vocalizations confirms differential patterns of lateralization. Whereas the left side of the face moves earlier and more during facial expressions of emotion (e.g., Hauser, 1993; Fernández-Carriba et al., 2002a,b), Losin et al. (2008) found that chimpanzees’ intentional communicative vocalizations were associated with greater movement on the right side of the face. They compared oro-facial asymmetries for emotional signals (pant-hoot, food-bark) and referential, communicative signals that the captive chimpanzees had learned to use to intentionally attract the attention of humans (raspberry, extended grunt). Results indicated that the emotional signals were expressed more intensely on the left side of the chimpanzees’ faces, but in striking contrast, the intentional communicative signals prompted greater movement in the right hemiface, suggesting left hemisphere control of learned oro-facial movements. This pattern of results was recently replicated in a second colony of captive chimpanzees (Wallez et al., 2012). As such, these findings suggest that just as humans show predominantly left lateralization for intentional communication (language) and right hemisphere lateralization for emotion, non-human primates like chimpanzees show left lateralization for intentional communication (learned, attention-getting sounds) and right hemisphere lateralization for emotion.

In addition, studies asking humans to judge the emotional expressivity of non-human primate faces confirm that we perceive the left hemiface as being more expressive. Hauser (1993) created left–left and right–right chimeric faces of rhesus macaques’ fear grimaces and asked human participants to indicate which appeared more emotionally expressive. The overwhelming majority of participants (41/43) deemed the left–left chimeras more emotionally expressive than the right–right chimeras, consistent with greater expressivity in the left side of the monkey’s faces as a consequence of predominant right hemisphere innervation. Similar findings have been reported for judgments of baboon (Wallez and Vauclair, in press) and chimpanzee chimeric faces (Fernández-Carriba et al., 2002a), with the consistent left–left chimera preference confirming right hemisphere dominance for the expression of emotion in non-human primates.

Although Hauser’s (1993) and Fernández-Carriba et al.’s (2002a,b) findings appear consistent with a right hemisphere model of emotional control, Hook-Costigan and Rogers’ (1998) marmoset research supports an alternate, valence-based conceptualization. According to *the valence hypothesis*, both hemispheres contribute to emotion processing: the right hemisphere is argued to control negative emotion, whereas the left hemisphere controls positive emotion (see Demaree et al., 2005, for review in humans). This valence-based asymmetry may reflect a more basic lateralization of behavioral tendencies to approach positive stimuli and avoid/withdraw from aversive stimuli (see Rutherford and Lindell, 2011, for review of approach/avoidance lateralization; Harmon-Jones, 2004, and Carver and Harmon-Jones, 2009, for detailed discussion of anger as an approach-related emotion). Hook-Costigan and Rogers reported greater expressivity on the left side of marmosets’ faces for expressions and vocalizations of fear, however the right side of the face was more expressive when marmosets made social contact calls. Consequently their data appear consistent with right hemisphere control of negative, and left hemisphere control of positive, emotion. To date these are the only non-human primate data supporting the valence hypothesis and until further consistent evidence is available, should be interpreted cautiously. It is interesting to note, however, that just as there is conflicting evidence supporting the right hemisphere and valence hypotheses in humans, the contention is mirrored in the non-human primate research. Indeed, some recent evidence fails to support either the right hemisphere or valence hypothesis, with Wallez and Vauclair (2012) reporting a right cheek (i.e., left hemisphere) bias for screaming in infant macaques.

Overall, however, the majority of research investigating the lateralization of emotional expression in non-human primates indicates right hemisphere dominance for emotion control. Data from investigations assessing species including macaques (e.g., Hauser, 1993), baboons (e.g., Wallez and Vauclair, 2011), and chimpanzees (e.g., Fernández-Carriba et al., 2002a,b), indicate that the left side of these non-human primates’ faces is more emotionally expressive, mobilizing earlier and moving more. Given this anatomical expressional asymmetry, it is not surprising that chimeras composed of two left chimpanzee hemifaces are overwhelming perceived as more emotionally expressive than right–right chimeras (e.g., Hauser, 1993). As such, the non-human primate findings appear consistent with those observed in human studies, indicating right hemisphere dominance for emotion control. Moreover, the fact that the expressional asymmetry is evident in Old World monkey species like baboons and macaques implies that right hemisphere specialization emerged early in primate evolution (at least 30–40 million years ago, Boyed and Silk, 2000) and was phylogenetically conserved in later-evolving primate species, such as chimpanzees and humans. Thus far from being an exclusively human trait, hemispheric lateralization for emotional expression is evident across primate species.

## Emotion Lateralization in Non-Human Primates: Perception

The ability to read others’ emotional expressions provides valuable information about affective state and allows an animal to modify its own behavior in light of the information gained. As such, the ability to accurately and efficiently distinguish facial emotions is a vital skill. Not surprisingly then, primates have evolved to develop exquisite mechanisms for recognizing emotions. For example, within a few days of birth, human babies can distinguish between happy, sad, and surprised expressions (e.g., Field et al., 1982), and by 6 months, human infants have gained the ability to distinguish between the negative emotions of fear, anger, and sadness (Serrano et al., 1992). Whilst there is a considerable body of research assessing facial emotion expression in non-human primates, research investigating facial emotion perception in non-human primates is rather more scarce. The lack of research in this area presumably reflects the challenges inherent in such an endeavor: while expressional asymmetries can be easily assessed using an observational paradigm, assessment of perceptual asymmetries may appear less amenable to naturalistic observation. That said, a number of researchers have investigated emotion perception in non-human primates, and these data are again consistent in supporting a greater role for the right hemisphere in emotion processing.

For example, when gelada baboons engage in agonistic behavior, they preferentially favor their left visual field (right hemisphere; Casperd and Dunbar, 1996). The authors assessed orientational asymmetries of male baboons during fights, threats, and approaches, and found that both members of a conflict pair used their left visual field more often than the right. As the authors write, “… an animal which orients its head so as to hold its opponent on the left side… does so in order to ensure that signal information is transmitted disproportionately to the right cerebral hemisphere,” (p. 58), consistent with right hemisphere dominance for emotion processing. Similar findings have been reported for approach behaviors in mangabeys, suggesting that the left visual field bias is not restricted to negative interactions in primates. Baraud et al. (2009) found that mangabeys (a type of Old World monkey) are more inclined to approach a conspecific on the left. Such an approach privileges visual access to the more expressive left side of the face, presumably facilitating efficient emotion communication.

Whereas Casperd and Dunbar (1996) and Baraud et al. (2009) used natural observational methods to assess emotion perception in non-human primates, other researchers have employed experimental paradigms, including split brain research. In humans, the split brain operation is performed to relieve intractable epilepsy; by severing the corpus callosum the two sides of the brain are functionally isolated, preventing epileptiform activity from traveling between the hemispheres (Sperry, 1968). Following split brain operations on 26 rhesus macaques, Vermeire and Hamilton (1998) trained the macaques to discriminate macaque faces on the basis of emotional expression. When faces were presented to the macaques’ isolated right hemispheres, emotion discrimination performance was significantly better than when the faces were presented to the isolated left hemispheres, indicating a right hemisphere advantage for the perception of facial emotion in Old World monkeys.

Research investigating emotion perception in chimpanzees similarly indicates a right hemisphere advantage. Parr and Hopkins (2000) showed six chimpanzees emotionally evocative videos depicting play (positive), scenery (neutral), and severe aggression (negative); whilst the chimpanzees watched the videos their tympanic membrane temperature (Tty) was recorded (Tty provides an indirect but reliable measure of brain temperature, indexing changes in autonomic and behavioral activity). Parr and Hopkins’ data indicted that right ear Tty increased for all chimpanzees when they were viewing the negative emotional video, consistent with greater right hemisphere involvement in processing negative emotion.

Importantly, this right hemisphere emotion perception bias is not restricted to the perception of emotion in conspecifics: chimpanzees also show a right hemisphere bias when perceiving facial emotion in humans (Morris and Hopkins, 1993). Morris and Hopkins (1993) trained three chimpanzees to discriminate between pairs of human chimeric faces on the basis of which chimera appeared happier (each chimera was composed of one neutral and one smiling half). During the test phase the researchers found that the chimpanzees were more likely to select the chimera with the smiling half falling in the left visual field (right hemisphere). Previous human research using an identical task similarly indicated a preference for chimeras with the emotion falling in the left visual field (Levy et al., 1983), indicating a high degree of consistency in the lateralization of emotion perception in these closely related primates.

In sum, the results of studies investigating the perception of emotion in non-human primates echo the results of studies assessing the expression of emotion in non-human primates, indicating right hemisphere dominance for emotion control. Although studies examining emotion perception in non-human primate species have investigated only a few species (i.e., macaques, baboons, chimpanzees), the data from those investigations indicate a left visual field (right hemisphere) bias for the perception of emotion in both conspecific (e.g., Vermeire and Hamilton, 1998) and human faces (e.g., Morris and Hopkins, 1993), and during natural interactions (Casperd and Dunbar, 1996; Baraud et al., 2009). Given that this bias is consistent across both Old World monkeys and great ape species, and is similarly evident in humans, it seems reasonable to suggest that just as the asymmetry for emotion expression is evident across primate phylogeny, a right hemisphere bias for emotion perception is conserved across primate species.

## Conclusion and Future Directions

Far from being a uniquely human trait, the research reviewed suggests that lateralization of function is a universal characteristic of primate species. In particular, the right hemisphere asymmetry that characterizes the expression and perception of emotion in humans appears to be pervasive across primate phylogeny. From Old World monkeys like baboons and macaques, to great apes and humans, species thought to have evolved from a shared ancestor over 30–40 million years ago (Stewart and Disotell, 1998; Boyed and Silk, 2000) show similar emotional asymmetries. Across primate species the right hemisphere’s dominance in emotion processing is manifest, leading to greater emotional expressivity in human and non-human primates’ left hemifaces (e.g., Borod, 1993; Fernández-Carriba et al., 2002a,b), and greater perceptual sensitivity to emotion in human and non-human primates’ left visual fields (controlled by the right hemisphere; e.g., Ley and Bryden, 1979; Morris and Hopkins, 1993). Given that these emotional asymmetries are present in Old World monkey species, the right hemisphere’s specialization for emotion processing is likely to have emerged early in primate evolution, with evidence implying phylogenetic conservation in later-evolving primate species, including humans. As such, the research reviewed strongly supports the notion of organizational continuity in the neural substrates supporting emotion processing in primate species.

This review has argued that the right hemisphere asymmetry for emotion reflects homology across primate species (i.e., results from shared primate ancestry), however it is important to note an alternate possibility. Hopkins and Cantalupo (2008) point out that continuity in patterns of asymmetry may result from homology but could alternately reflect homoplasy: convergent evolution of common patterns of asymmetry that evolved independently. However, given that the patterns of lateralization in lower and higher primates are conserved despite marked changes in the organization of sensory systems (e.g., vision), it appears probable that the consistency in patterns of primate lateralization reflects homology (see Hopkins and Cantalupo, 2008, for discussion).

Beyond the suggestion of continuity in emotion lateralization for human and non-human primates, this review makes it strikingly apparent that there are significant gaps in the non-human primate literature. Whilst emotional expression has been subject to comparatively greater investigation than emotion perception in non-human primates, even there the studies are restricted to only a few species (i.e., macaques, baboons, chimpanzees, marmosets). Whether the right hemisphere’s dominance for emotion extends to prosimians remains an open question. Though it appears probable that the left side expressivity bias seen in Old World monkeys, New World Monkeys, great apes, and humans will be similarly evident in their more phylogenetically distant primate relatives, only investigation of facial displays in more primitive primates, such as lemurs, galagos, and lorises, will resolve the question.

As Ward (1991) notes, examination of prosimian species offers unique opportunities in the investigation of the evolution of primate lateralization: prosimians are less complex than anthropoid primates in terms of behavior and brain structure, yet being primates, can serve as models of human laterality. Given that prosimians, such as galagos, are thought to retain many of the characteristics ancestral to those of all living primates (Brothers, 1990), examination of their facial expressions may help shed light on the degree to which expressional asymmetries are likely to have developed over the course of primate evolution. Moreover, prosimians present in the potentially unique position of serving as a bridge between non-primate mammals and anthropoid primates. Research investigating emotion lateralization in non-primate mammals such as dogs (e.g., Quaranta et al., 2007; Siniscalchi et al., 2010) and horses (e.g., De Boyer Des Roches et al., 2008; Farmer et al., 2010) offers evidence indicating the lateralization of emotion perception in these species; however studies assessing asymmetries in the expression of facial emotion in non-primate mammal have yet to be conducted. Should examination of prosimian species confirm hemifacial asymmetries in emotion expression, assessment of asymmetries in the facial expressions of non-primate mammals appears a logical next step.

Investigation of emotion lateralization in the owl monkey family *Aotus* (also known as the night monkey) also offers intriguing possibilities. Being a nocturnal anthropoid, this group of non-human primates has evolved to possess comparatively less differentiated facial musculature than diurnal primates and is reported to have virtually no facial expressions (Huber, 1931; Chevalier-Skolnikoff, 1973). Examination of both expressional and perceptual asymmetries in this family thus affords a unique opportunity to assist in determining the extent to which nature and nurture shape patterns of emotion lateralization in primates. By examining the magnitude of the emotion asymmetry present in the nocturnal *Aotus* and comparing it with that seen in a similar but diurnal (e.g., *Cebidae*) or cathmeral anthropoid species (e.g., *Aotus azarae azarae* which is sporadically active during the day and night), one may speculate on the influence of experience on emotion lateralization. Observation of others’ emotion expressions may increase the magnitude of the hemispheric asymmetry for emotion processing in diurnal primates via experience-dependent processes; such influences may be less likely to induce changes in nocturnal and cathmeral primates like *Aotus*.

The fact that hemispheric asymmetries are evident across primate species has implications beyond the lateralization of emotion. Where once lateralization was thought to be a defining human attribute (e.g., Pruner-Bey, 1865), the studies reviewed indicate that emotion lateralization is the rule rather than the exception among primates, confirming that the emergence of hemispheric asymmetry was independent of language. Whilst theorists seek to distinguish uniquely human characteristics (mooting language, tool use, and creativity as likely contenders), it appears increasingly apparent that such anthropocentric goals are of limited utility; human and non-human primates are far more similar than we are different. The research reviewed indicates that the right hemisphere asymmetry for emotion processing is pervasive from Old World monkeys to chimpanzees and humans, and future research will determine whether this pattern of lateralization similarly extends to more distantly related prosimians. If right hemisphere emotion lateralization is confirmed in prosimians, investigation of lateralization in non-primate mammals offers a logical next step in the journey toward understanding the evolution of emotion lateralization.

## Conflict of Interest Statement

The authors declare that the research was conducted in the absence of any commercial or financial relationships that could be construed as a potential conflict of interest.
